# London Protocol under water-perfused HRM in a healthy population, towards novel 3D manometric parameters in an evaluation of anorectal functional disorders

**DOI:** 10.1186/s12876-024-03207-w

**Published:** 2024-04-04

**Authors:** Alexandre Anefalos, Carlos Augusto Real Martinez, Claudio Saddy Rodrigues Coy

**Affiliations:** Department of Surgery, FCM, State University of Campinas-UNICAMP, Campinas, SP Brazil

**Keywords:** High-resolution manometry (HRM), 3D high-resolution manometry, Anorectum, Water-perfused, Healthy volunteers

## Abstract

**Background/Aim:**

London Protocol (LP) and Classification allied to high-resolution manometry (HRM) technological evolution has updated and enhanced the diagnostic armamentarium in anorectal disorders. This study aims to evaluate LP reproducibility under water-perfused HRM, provide normal data and new parameters based on 3D and healthy comparison studies under perfusional HRM.

**Methods:**

Fifty healthy (25 F) underwent water-perfused 36 channel HRM based on LP at resting, squeeze, cough, push, and rectal sensory. Additional 3D manometric parameters were: pressure-volume (PV) 10^4^mmHg^2^.cm (resting, short and long squeeze, cough); highest and lowest pressure asymmetry (resting, short squeeze, and cough). Complementary parameters (CP) were: resting (mean pressure, functional anal canal length); short squeeze (mean and maximum absolute squeeze pressure), endurance (fatigue rate, fatigue rate index, capacity to sustain); cough (anorectal gradient pressure); push (rectum-anal gradient pressure, anal canal relaxation percent); recto-anal inhibitory reflex (anal canal relaxation percent).

**Results:**

No difference to genders: resting (LP, CP, and 3D); short squeeze (highest pressure asymmetry); endurance (CP); cough (CP, highest and lowest pressure asymmetry); push (gradient pressure); rectal sensory. Higher pressure in men: short squeeze (maximum incremental, absolute, and mean pressure, PV, lowest pressure asymmetry); long squeeze (PV); cough (anal canal and rectum maximum pressure, anal canal PV); push (anal canal and rectum maximum pressure). Anal canal relaxation was higher in women (push).

**Conclusions:**

LP reproducibility is feasible under water-perfused HRM, and comparative studies could bring similarity to dataset expansion. Novel 3D parameters need further studies with healthy and larger data to be validated and for disease comparisons.

**Key points:**

• London Protocol and Classification allied with the technological evolution of HRM (software and probes) has refined the diagnostic armamentarium in anorectal disorders.

• Novel 3D and deepening the analysis of manometric parameters before the London Classification as a contributory diagnostic tool.

• Comparison of healthy volunteers according to the London Protocol under a perfusional high-resolution system could establish equivalence points.

**Supplementary Information:**

The online version contains supplementary material available at 10.1186/s12876-024-03207-w.

## Introduction

London Protocol has emerged as a landmark in the diagnostic of anorectal disorders, proposing technical standardization and a novel manometric classification based on a hierarchical division of findings [1, 2]. As backdrop, high-resolution manometry (HRM) providing a simultaneous and dynamic view of anorectal physiology, combining high sensor density, and minimizing movement artifacts with intuitive three-dimensional topographical color plots, has constituted an irrefutable advance diagnostic tool [3, 4].

Several studies have aimed to determine normal values of anorectal HRM and to demonstrate equivalent manometric findings, however, under a range of variables. Distinct pressure-sensing transducer systems, solid state or water-perfused, in high-resolution or high-definition, combined with different circumferential settings probes, in distribution and sensors number [4, 5], whose results also affected by non-standard technical procedures [5, 6].

This study aims to evaluate 50 healthy volunteers according to the London Protocol under water-perfused 36-channel HRM. Our findings were compared with other studies of perfusional high-resolution systems, as well as deepen the analysis of manometric parameters before the London Classification benchmark and propose novel 3D parameters that can be a contributory diagnostic tool.

## Materials and methods

### Subjects

Healthy, asymptomatic 50 subjects were consecutively recruited at the Center of Physiology of Piracicaba (São Paulo, Brazil) from February 2022 to December 2022. We studied 25 men, mean age of 41.27± 8.48 years (age range: 22-60 years) and mean body mass index (BMI) of 26.33± 3.58, and 25 women, mean age of 45.4±18.38 (age range: 18-60 years) and mean BMI of 27.04±4.72. We did not have volunteers older than 60 who did not meet the exclusion criteria. Regarding the obstetric history, 68% delivered (88% cesarean section, 12% no forceps vaginal delivery), 41.18% primipara, and 58.8% multipara.

Inclusion criteria included healthy volunteers’ men and women from 18 years old with Bristol Stool Form Scale (BSFS) type 4, daily bowel movement frequency and no use of laxatives. Exclusion criteria were: (1) previous anorectal surgery; (2) diagnosis of anorectal functional disorders according to the Rome IV criteria; (3) current or past anorectal disease (inflammatory bowel disease, hemorrhoids, fissures, fistulas, or neoplasms); (4) history of pelvic or obstetric trauma and (5) previous radiotherapy.

### Ethics

The study protocol was approved by the Research Ethics Committee of State University of Campinas (UNICAMP- São Paulo, Brazil). Informed consent was obtained of all participants and no identifiable data present. There is no conflict of interest. All authors contributed sufficiently to be named as authors and are responsible for the manuscript. No professional or ghostwriter was hired.

### Equipment

All patients underwent 36-channel water-perfused HRM (Multiplex Alacer Biomédica, São Paulo, Brazil). An internal pump of the equipment maintains a constant flow rate of 0.3ml per minute (min) of sterile water. The polyvinyl chloride (PVC) probe used has an external diameter of 4.7mm, incorporating 36 pressure channels arranged radially spanning 6cm. At first 4cm, 28 sensors with 7 channels spaced radially apart 51.4° (2.1mm) and 1 cm axially. In the last 2 cm, 8 sensors with 4 channels spaced radially 90° (3.7mm). Four cm from the distal channels group was placed a 5 cm latex balloon communicated with central lumen. The probe fulfilled with fluid was calibrated to the software and the sensors were zeroed at the level of the external anal orifice and at 36.7mmHg (50cm of water) in its upper limit before each exam. The topographic color plot of manometric pressure data, 3D vectors, and respective pressure volumes (PV) were acquired via the dedicated commercial software (Alacer Biomédica, São Paulo, Brazil).

### Study protocol

All volunteers performed anorectal preparation the night before the exam with a 4.0 g glycerin suppository. The subjects were informed about all steps presented in London Protocol (LP) in a quiet room and positioned in left lateral decubitus with hips and knees flexed at 90°. A lubricated probe was gently placed in the rectum with the first set of sensors checked at the internal anal orifice. Three-minute stabilization period was observed before test maneuvers.

The sequence and manometric parameters evaluated were:

- Rest over 60 seconds (s) - LP: mean maximum pressure (mmHg); complementary parameters: mean pressure (mmHg), functional anal canal length (cm); 3D manometric parameters: resting PV (10^4^mmHg^2^.cm), highest and lowest pressure asymmetry (%).
- Short squeeze (3 squeezes lasting 5 s separated by 30 s between them) - LP: maximum incremental pressure squeeze (mmHg); complementary parameters: mean pressure (mmHg), maximum absolute squeeze pressure (mmHg); 3D manometric parameters: short squeeze PV (10^4^mmHg^2^.cm); highest and lowest pressure asymmetry (%).
- Long (endurance) squeeze (sustained voluntary effort over 30 s) -complementary parameters: fatigue rate (mmHg), fatigue rate index (min), and capacity to sustain (%); 3D manometric parameters (10^4^mmHg^2^.cm): 1/3; 2/3 and 3/3 long squeeze PV.
- Cough (2 single coughs separated by 30 s between them) - LP: maximum pressure anal canal (mmHg), maximum pressure rectum (mmHg); complementary parameter: anorectal gradient pressure (mmHg); 3D manometric parameters: anal canal PV (10^4^mmHg^2^.cm) in cough, highest and lowest pressure asymmetry of the anal canal (%).
- Push (15 s duration, separated by 30 s between them) - LP: maximum pressure anal canal (mmHg), maximum pressure rectum (mmHg); complementary parameters: rectum-anal gradient pressure (mmHg), anal canal relaxation percent (%).
- Recto-anal inhibitory reflex (RAIR- performed with 50mls, 30 s recovery interval) - complementary parameter: anal canal relaxation percent (%).
- Rectal sensory test (1-5 ml/s rate manually controlled) - LP: first sensation volume (ml), desire to defecate volume (ml), maximum tolerated volume (ml).

#### Endurance complementary parameters

The fatigue rate (FR) was generated by a linear regression model measuring the pressure decrease (mmHg) per min [7]. The computerized calculation of fatigue rate index (FRI) in minutes, according to Marcello et al. [7, 8], was derived by the formula: [(maximal) squeeze pressure - resting pressure (mmHg)] /- FR (mmHg/min).

Capacity to sustain (CS) calculation was based on a linear regression model adapted from Saad et al. [9] and expressed the percentage over 30 s endurance to maintain an increase in anal pressure > 50% of maximum squeeze (previous article: increase > 70% over 40 s) with the formula: [100 x (maximum regression squeeze pressure + FR x 0.5)]/maximum regression squeeze pressure.

#### 3D manometric parameters

**Fig. 1:**
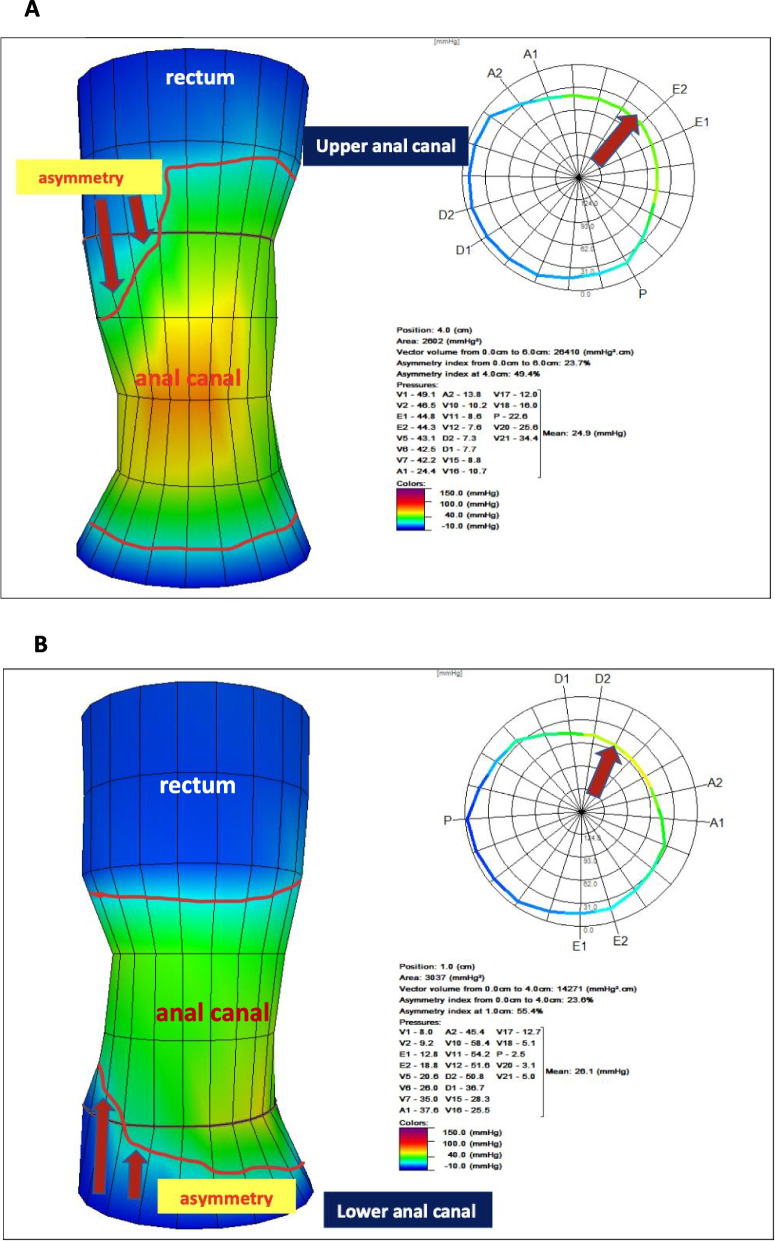
Example of resting PV. The red arrows show the area of asymmetry in the vectorgram at the upper and lower landmarks of the functional anal canal. Upper asymmetry (**a**) and lower asymmetry (**b**) are shown

The 3D pressure-volume was generated by the software in the 6 cm of probe sensors. The upper and lower references in centimeters to determine the vectorgram of the functional anal canal were performed manually, establishing a measurement above 30mmHg as a considerable value. In the asymmetry of the colorimetric contour, evaluating the upper anal canal, when over 50% of the area filled in the corresponding vectorgram, the upper centimeter was considered as reference, and when below 50%, the corresponding lower centimeter was selected. In the asymmetry of the lower anal canal, when over 50% of the filling of the area in the corresponding vectorgram, the inferior centimeter was marked and below 50%, the superior one (Fig. 1). We simplified the 3D findings to 10^4^mmHg^2^.cm for comparative analyses.

The 3D pressure-volume analyzed in subsequent steps of the London Protocol, resting, short squeeze, long squeeze separated by 3 periods of 10 s and cough are depicted in Supplementary Figures (SF1- SF3).

### Statistical analysis

Shapiro-Wilk’s method was used to assess normal distribution of data. The unpaired t-test was applied to the difference between normally distributed parameters and the Mann-Whitney U test under univariate analysis for non-normal data. Variables are expressed as mean (standard deviation) and median (interquartile range). The entire normality study database also including range, minimum (min), maximum (max); 95% confidence interval (95% CI), 5th and 95th percentiles are depicted in Supplementary Tables (ST). All *p*< 0.05 were considered significant. Statistical analyses were performed using the R software version 4.2.2.

Descriptive statistics (mean and median) were used to compare studies of normality under water-perfused HRM.

## Results

High-resolution manometric values of 50 healthy volunteers are divided by analysis steps according to the London Protocol (LP), expressed and compared by gender as follows: resting, short and long squeeze, cough, push, rectal sensory threshold parameters, and RAIR.

Resting manometric analysis (LP, complementary and 3D parameters) showed no statistically significant difference between genders (Table 1 and ST1-ST2). Evaluating the mean asymmetry of the functional anal canal, based on 3D analysis, we found for all 50 healthy volunteers, for the highest pressure asymmetry, 26.8% ±9.6, and for the lowest pressure asymmetry, 25.1% ±11,6.

**Table 1:** Resting manometric parameters comparing 25 healthy (female x male)

**Resting manometric parameters**	**Female (25)** **Mean (SD) Med IQR)**		**Male (25)** **Mean (SD) Med(IQR)**		***p***
**London Protocol parameter**					
**Mean maximum pressure (mmHg)**	61.1 (16.3)	59.9 [51.9;74.0]	62.8 (14.7)	60.9 [50.7;75.3]	0.71
**Complementary parameters**					
** Mean pressure** **(mmHg)**	38.4 (9.6)	38.9 [30.7;46.0]	42.1 (9.2)	40.0 [35.4;45.1]	0.20
** Functional anal canal length (cm)**	3.5 (1.0)	3.6 [2.7;4.4]	3.9 (0.8)	3.9 [3.3;4.6]	0.14
**3D parameters**					
** Resting PV (10** ^**4**^ **mmHg** ^**2**^ **.cm)**	2.4 (1.5)	2.0 [1.1;3.3]	2.9 (1.6)	2.6 [1.7;3.6]	0.31
** Highest pressure asymmetry (%)**	26.3 (9.8)	23.9 [19.3;29.9]	27.3 (9.6)	26.8 [21.1;32.5]	0.72
** Lowest pressure asymmetry (%)**	27.1 (13.0)	25.4 [19.4;32.4]	23.2 (10.0)	22.6 [17.5;28.2]	0.31

*SD* Standard deviation, Med Median, *IQR* Interquartile range, *PV* Pressure-volume

Squeeze manometric parameters (Table 2 and ST3-ST4) showed higher pressure in men in the analysis of the maximum incremental pressure squeeze (p < 0.05), mean and maximum absolute squeeze pressure (*p*<0.01), and in the 3D parameters, short and long squeeze PV 1/3 and 2/3 (*p*<0.01), and endurance PV 3/3 (*p*< 0.05). No statistically significant difference was observed compared to genders analyzing complementary parameters to endurance: FR (*p*=0.64), FRI (*p*=0.42), and CS (*p*=0.24).

**Table 2:** Squeeze manometric parameters comparing 25 healthy (female x male)

Squeeze manometric parameters	Female (25) Mean (SD) Med (IQR)		Male (25)		***p***
			Mean (SD)	Med (IQR)	
**London Protocol parameters**					
**Maximum incremental pressure squeeze (mmHg);** short squeeze	108.7 (42.7)	102.5 [83.6;126.2]	143 (50.5)	146.3 [114.0;168.8]	**<0.05**
**Complementary parameters**					
**Mean pressure (mmHg);** short squeeze	117.0 (40.6)	107.8 [99.6;138.0]	155.1 (39.4)	157.2 [131.6;177.6]	**<0.01**
**Maximum absolute squeeze pressure (mmHg);** short squeeze	169.9 (44.0)	168.5 [142.8;183.5]	205.7 (45.2)	211.6 [180.0;239.6]	**<0.01**
**Fatigue rate (mmHg);** long squeeze	-74.9 (46.4)	-69.8 [-91.8; -47.5]	-60.5 (77.5)	-71.3 [-95.4; -13.8]	0.64
**Fatigue rate index (min);** long squeeze	1.6 (2.2)	0.8 [0.6;1.5]	2.0 (4.2)	1.0 [0.7;1.5]	0.42
**Capacity to sustain (%);** long squeeze	71.60 (14.8)	73.4 [61.0;80.0]	79.80 (22.1)	74.2 [66.9;93.6]	0.24
**3D parameters**					
**Short squeeze PV (10**^4^**mmHg².cm)**	21.0 (13.1)	18.8 [13.5;23.9]	36.2 (16.4)	30.9 [26.0;49.9]	**<0.01**
**Highest-pressure asymmetry (%); short squeeze**	15.8 (5.6)	16.0 [10.6;18.1]	14.1 (4.9)	13.5 [10.4;17.2]	0.27
**Lowest pressure asymmetry (%); short squeeze**	18.8 (9.2)	17.2 [11.4;23.3]	12.9 (4.5)	12.6 [9.7;15.1]	**<0.05**
**Long squeeze PV (1/3) 10**^4^**mmHg².cm**	11.8 (7.9)	9.9 [7.7;13.7]	19.0 (10.5)	15.4 [12.3;24.8]	**<0.01**
**Long squeeze PV (2/3) 10**^4^**mmHg².cm**	9.9 (7.2)	7.7 [4.4;13.1]	16.4 (10.5)	12.2 [9.1;24.7]]	**<0.01**
**Long squeeze PV (3/3) 10**^4^**mmHg².cm**	8.3 (5.6)	7.2 [4.0;11.3]	15.1 (10.7)	11.0 [9.1;20.5]	**<0.05**

*SD* Standard deviation, *Med* Median, IQR Interquartile range, *PV* Pressure-volume

Bold values indicate statistically significant (*p* < 0.05)

Regarding short squeeze symmetry, no difference was observed between genders for the highest pressure asymmetry with 14.9% ±5.3 for all 50 volunteers, however, the lowest pressure asymmetry was higher in females with 18.8±9.2, versus males, 12.9±4.5 (*p*<0.05).

Evaluating cough manometric parameters (Table 3 and ST5-ST6), the pressure was higher in men analyzing the maximum pressure anal canal (*p*< 0.05), the anal canal PV (*p*< 0.01), as well as the maximum pressure rectum (*p*<0.01). Complementary parameters (anorectal gradient pressure) and 3D parameters (highest and lowest pressure asymmetry) did not show differences between genders.

**Table 3:** Cough manometric parameters comparing 25 healthy (female x male)

Cough manometric parameters	Female (25)		Male (25)		***p***
	Mean (SD)	Med (IQR)	Mean (SD)	Med (IQR)	
**London Protocol parameters**					
**Maximum pressure anal canal (mmHg)r**	132 (31.6)	134.6 [127.6;155.0]	150.7 (28.8)	150.2 [124.9;171.6]	**<0.05**
**Maximum pressure rectum (mmHg)**	65.5 (23.8)	65.0 [47.0;79.3]	87.0 (27.3)	82.2 [72.5;95.5]	**<0.01**
**Complementary parameters**					
**Anorectal gradient pressure (mmHg)**	67.7 (22.1)	62.6 [48.6;83.9]	63.7 (26.8)	63.1 [55.7;71.9]	0.57
**3D parameters**					
**Anal canal PV in cough (10**^4^**mmHg².cm)**	11.0 (4.8)	10.6 [8.3;14.0]	15.1 (5.2)	15.3 [11.6;18.2]	**<0.01**
**Highest pressure asymmetry (%); anal canal**	14.4 (5.9)	13.9 [10.0;16.9]	16.0 (6.7)	14.9 [10.8;20.3]	0.39
**Lowest pressure asymmetry (%); anal canal**	14.7 (4.9)	14.9 [12.6;18.1]	12.5 (4.0)	11.7 [8.9;15.0]	0.08

*SD* Standard deviation, *Med* Median, IQR Interquartile range),*PV* Pressure-volume

Bold values indicate statistically significant (*p* < 0.05)

Push manometric parameters (Table 4 and ST7-ST8) showed higher pressure in men regarding to maximum pressure anal canal (*p*<0.05) and maximum pressure rectum (*p*<0.01). The anal canal relaxation (%) was higher in women (*p*<0.05). No difference in gender in rectum-anal gradient pressure.

**Table 4:** Push manometric parameters comparing 25 healthy (female x male)

Push manometric parameters	Female (25)		Male (25)		***p***
	Mean (SD)	Med (IQR)	Mean (SD)	Med (IQR)	
**London Protocol parameters**					
**Maximum pressure anal canal (mmHg)**	47.4 (19.0)	43.9 [35.7;53.9]	61.3 (26.5)	51.3 [46.7;71.1]	**<0.05**
**Maximum pressure rectum (mmHg)**	27.9 (20.5)	23.9 [19.0;33.2]	42.5 (18.5)	34.9 [31.1;54.1]	**<0.01**
**Complementary parameters**					
**Rectum-anal gradient pressure (mmHg)**	-16.1 (22.5)	-17.9 [-24.2; -1.5]	-22.9 (13.2)	-20.2 [-31.4; -13.9]	0.20
**Anal canal relaxation percent (%)**	6.4 (36.0)	10.2 [-22.0;36.4]	-23.7 (54.1)	-5.7 [-48.8;10.2]	**<0.05**

*SD* standard deviation, *Med* Median, *IQR* Interquartile range

Bold values indicate statistically significant (*p* < 0.05)

Rectal sensory thresholds (first sensation volume, desire to defecate volume, and maximum tolerated volume) and RAIR (anal canal relaxation, %) did not show differences comparing men and women (Table 5 and ST9-ST10).

**Table 5:** Rectal sensory thresholds and RAIR parameters comparing 25 healthy (female x male)

**Rectal sensory thresholds parameters**	**Mean (SD)**	**Med (IQR)**	**p**
**London Protocol parameters**			
** First sensation volume (ml)**			
**Female**	22.9 (17.9)	16.0[10.0;32.0]	0.35
**Male**	20.7 (20.4]	12.0[6.0;22.0]	
** Desire to defaecate volume(ml)**			
**Female**	38.5 (19.7)	34.0[23.0;48.0]	0.77
**Male**	39.2 (24.0)	34.0 [20.0;50.0]	
** Maximum tolerated volume(ml)**			
**Female**	141.4 (53.1)	132.0[106.0;164.0]	0.57
**Male**	133.4 (44.3)	125.0[100.0;140.0]	
**Complementary parameters**			
** Anal canal relaxation (%)**			
**Female**	36.8 (15.8)	33.3 [25.7;44.9]	0.70
**Male**	38.4 (13.9)	39.4 [29.5;45.4]	

*SD* Standard deviation, *Med* Median, *IQR* Interquartile range

## Discussion

The technological evolution of anorectal manometry under the advent of high resolution combined with the London classification and protocol, similarly to the Chicago Classification [10], has provided in recent years the search for the homogenization of analysis metrics, however, based mostly on studies with solid-state equipment and probes.

The results of our study, reproducing the London Protocol, demonstrate the feasibility and possibility of seeking new metrics to expand the diagnostic armamentarium, under water perfused HRM system, reachable to many countries.

### 3D manometric parameters

The integrated pressurized volume (IPV), using rectum-anal spatiotemporal plot (amplitude, distance, and time) was studied to predict balloon expulsion time (BET) and dyssynergic defecation showing effectiveness [11] thus, searching for metric similarity (mmHg.s.cm) to distal contractile integral (DCI) on the update on esophageal HRM [12]. Corroborating, the 3D pressure-volume analysis of our study (resting, squeeze, and cough), based on the London Protocol, expressed in mmHg^2^.cm, can allow an easier, wider, and more intuitive assessment of the entire anal canal area (mmHg^2^) in its functional length (cm) and asymmetry, compared to the traditional pressure assessment (mmHg). Furthermore, a more accurate topographic representation of the anal canal pressure gradient in 3D has been highlighted for some research in pediatrics compared to 2D manometry which is usually based on a mean pressure [13].

Evaluating 3D pressure-volume in healthy, our study showed no difference between genders at resting (*p*=0.31) as well as mean maximum pressure obtained by LP (*p*=0.71). Analyzing 3D short squeeze and cough parameters, we found a statistically significant difference (*p*<0.01), higher in men, also evidenced in the respective manometric findings by LP, maximum incremental pressure, and maximum pressure in the anal canal (*p*< 0.05).

In addition, we also present a new long squeeze parameter to evaluate incontinence patients, separating into 3 analysis periods of 10 s, using 3D pressure-volume, which can bring more accurate analysis and help to refine the biofeedback therapy, with a statistically significant difference between genders in all periods: 1/3 and 2/3 (*p*<0.01), and 3/3 (*p*<0.05). Comparatively, the complementary manometric parameters findings to endurance, FR (mmHg), FRI (min), and CS (%), showed no differences comparing males and females.

Pressure asymmetry along the axial and circumferential on manometry has been described for healthy adults and children [10, 14]. Furthermore, the assessment of normal asymmetry values within the anal canal (rest and squeeze) and their respective quadrants on 3D HRM can contribute to the investigation of internal and external anal sphincter defects [15], especially when endoanal ultrasound, the gold standard, is not available, with a slight agreement reported by few studies comparing the two methods [15]. Nevertheless, normative data on asymmetry pressure in healthy to determine the functional impact of defects seen under 3D HRM, especially in incontinent patients, are still lacking [16].

We demonstrated our findings of resting and short squeeze anal canal asymmetry in healthy based on 3D HRM and LP, differentiating for the highest pressure, with no difference comparing genders, and to lowest pressure, which we found higher asymmetry in female evaluating squeeze (18.8% ±9.2). Jorge et al. [17] using an 8-channel conventional manometry vectorgram evaluated the highest pressure asymmetry and found no difference to genders at rest, similar to our study, although with different findings (7.2% ±2.3 vs 26.8% ±9.6) however, differently of our results, in short squeeze, obtained higher asymmetry in female (7.1% ±2.5). We did not find HRM studies, based on LP protocol or not, with normal asymmetry range values in healthy adult volunteers for comparison.

Regarding push maneuvers, we did not explore 3D parameters as useful markers to differentiate pelvic dyssynergia or predict BET as studied with IPV [11], due to the imprecise delimitation of component extension of the rectal ampulla and anal canal and their 3D manometric dynamic interactions found, constituting a gap of our results. Our complementary parameters findings showed negative values of rectum-anal gradient without rectal distension and no difference to gender, and evaluating anal canal relaxation we found in the men group a lowest and negative value (*p*<0.05), whose potential explanations have been discussed for previous studies [6, 18], emphasizing that the search for another manometric marker to constipated seems relevant.

Our study has some limitations, such as the relatively small sample size, to a certain extent due to the COVID-19 pandemic social contact restrictions, especially enforced in healthcare facilities, and the lack of stratification of analyzes by age group, parity, or body mass index. The novel 3D parameters findings (10^4^mmHg^2^.cm) presented based on pressure-volume, according to the LP standardization steps, as well as the anal canal asymmetry findings, need further studies with larger dataset evaluating healthy volunteers to be validated as well as for disease comparisons, especially incontinent patients, to determine the real impact as a contributory diagnostic tool.

### Studies comparison of healthy volunteers under perfusional high-resolution system

The comparison of water-perfused HRM manometric parameters studies [19–21] by gender and separated by analyzes (rest, squeeze, cough, push, rectal sensory thresholds, and RAIR) are depicted in Table 6.

**Table 6:** Comparison of water-perfused HRM manometric parameters studies

Manometric parameters	Present article (channel = 36) London Protocol	Deshmukh et al.^**19**^ (channel = 20)	Viebig et al.^**20**^ (channel = 24)	Rasijeff et al.^**21**^ (channel = 10)
**Resting manometric parameters**				
**Mean maximum pressure(mmHg)**				
** Female**	n = 25	n = 29	n = 30	n = 40
Mean(SD)	61.1(16.3)			64.0
Med(IQR)	59.9[51.9;74.0]	94.0[48.0;117.0]		
5th; 95th	[31.9;77.9]			[34.0;101.0]
** Male**	n = 25	n = 64	n = 20	n = 20
Mean (SD)	62.8 (14.7)			67.0
Med (IQR)	60.9[50.7;75.3]	88.0[33.0;132.0]		
5th; 95th	[43.0; 86.4]			[40.0;116.0]
**Mean pressure (mmHg)**				
** Female**				
Mean (SD)	38.4(9.6)		79.8(4.0)	
Med IQR)	38.9[30.7;46.0]			
** Male**			72.20(3.0)	
Mean (SD)	42.1 (9.2)			
Med (IQR)	40.0[35.4;45.1]			
**Functional anal canal length (cm)**				
** Female**				
Mean(SD)	3.5(1.0)		3.0(0.1)	
Med(IQR)	3.6[2.7;4.4]	1.5[1.0;3.2]		
** Male**				
Mean (SD)	3.9 (0.8)		3.3(0.1)	
Med (IQR)	3.9[3.3;4.6]	2.5[1.1;3.8]		
**Squeeze manometric parameters**				
**Maximum incremental pressure(mmHg)**				
** Female**				
Mean(SD)	108.7(42.7)			105.0
Med(IQR)	102.5[83.6;126.2]	66.0[10.0;160.0]		
5th; 95th	[64.5;178.8]			[27.0;188.0]
** Male**				
Mean (SD)	143(50.5)			177.0
Med (IQR)	146.3[114.0;168.8]	90.0[32.0;150.0]		
5th; 95th	[64.7;216.2]			[36.0;305.0]
**Maximum absolute squeeze pressure(mmHg)**				
** Female**				
Mean(SD)	169.9 (44.0)		170.7(8.0)	
Med(IQR)	168.5[142.8;183.5]	147.0[83.0;259.0]		
** Male**				
Mean (SD)	205.7(45.2)		229.50(17.0)	
Med (IQR)	211.6[180.0;239.6]	165.0[90.0;377.0]		
**Cough manometric parameters**				
**Maximum incremental pressure(mmHg)**				
** Female**				
Mean(SD)				79.0
Med(IQR)				
5th; 95th				[28.0;136.0]
** Male**				
Mean (SD)				91.0
Med (IQR)				
5th; 95th				[29.0;152.0]
**Push manometric parameters**				
**Maximum pressure anal canal(mmHg)**				
** Female**				
Mean(SD)	47.4 (19.0)			
Med(IQR)	43.9[35.7;53.9]	63.0[18.0-100.0]		
** Male**				
Mean (SD)	61.3 (26.5)			
Med(IQR)	51.3[46.7;71.1]	82.0[36.0-170.0]		
**Maximum pressure rectum(mmHg)**				
** Female**				
Mean(SD)	27.9(20.5)			
Med(IQR)	23.9[19.0;33.2]	54.0[26.0-117.0]		
** Male**				
Mean (SD)	42.5 (18.5)			
Med (IQR)	34.9 [31.1;54.1]	70.0[34.0-133.0]		
**Rectum-anal gradient pressure(mmHg)**				
** Female**				
Mean(SD)	-16.1(22.5)			
Med(IQR)	-17.9[-24.2; -1.5]	6.0[-39.0;51.0]		
** Male**				
Mean (SD)	-22.9 (13.2)			
Med (IQR)	-20.2[-31.4; -13.9]	-6.0[-78.0;66.0]		
**Anal canal relaxation (%)**				
** Female**				
Mean(SD)	6.4 (36.0)			
Med(IQR)	10.2[-22.0;36.4]	42.0[-24.0;80.0]		
** Male**				
Mean (SD)	-23.7(54.1)			
Med (IQR)	-5.7[-48.8;10.2]	16.0[-38.0;53.0]		
**Rectal sensory thresholds parameters and RAIR**				
**First sensation volume(ml)**				
** Female**				
Mean(SD)	22.9(17.9)		31.0(1.2)	
Med(IQR**)**	16.0 [10.0;32.0]	30.0 [10.0;80.0]		
** Male**				
Mean(SD)	20.70(20.40]		43.0(4.8)	
Med(IQR)	12.0[6.0;22.0]	40.0[10.0;170]		
**Desire to defaecate volume(ml)**				
** Female**				
Mean(SD)	38.5(19.70)		100.0(7.0)	
Med(IQR)	34.0[23.0;48.0]	90.0[50.0;170.0]		
** Male**				
Mean(SD)	39.20(24.0)		102.0(9.4)	
Med(IQR)	34.0 [20.0;50.0]	105.0[40.0;250.0]		
**Maximum tolerated volume(ml)**				
** Female**				
Mean(SD)	141.4(53.10)		162.0(10.9)	
Med(IQR)	132.0[106.0;164.0]	140.0[80.0;270.0]		
** Male**				
Mean(SD)	133.4(44.3)		167.0(9.8)	
Med(IQR)	125.0[100.0;140.0]	160.0[80.0;310]		
**Anal canal relaxation (%)**				
** Female**				
Mean(SD)	36.8 (15.8)			
Med(IQR)	33.3 [25.7;44.9]	44.0[17.0;80.0]		
** Male**				
Mean(SD)	38.4 (13.9)			
Med(IQR)	39.4 [29.5;45.4]	49.5[0.0;87.0]		

*SD* Standard deviation, *Med* Median, *IQR* Interquartile range, n volunteers

Most published research have utilized solid-state (SS) HRM [10]. SS HRM advantages over perfusional HRM have been highlighted supported by greater sensor sensitivity to rapid pressure change thus emphasizing the distinction of normality manometric values and consequently, they should not be interchangeable between different equipment and catheters [21]. Furthermore, despite the time proposed by LP, 10 to 12 minutes for the procedure, the presence of local wetness, inherent for perfusional system, as well as the number of sensors and water infusion rate (ml/min) used, must also be considered in the results obtained.

Comparing healthy volunteers’ studies under a perfusional high-resolution system, we aimed to find equivalent points and similarities comparable to relevant solid-state studies, which may contribute to the anorectal scientific research scenario. The paper comparisons used consolidated descriptive statistics, means and respective confidence intervals (5th and 95th percentiles), medians, and interquartile range, depending on the data presented. However, for interpretive homogenization, we found some methodological differences from the London Protocol and data acquisition that may have influenced the comparison as well as different channel probes (10 to 36) and samples. In addition, more advanced statistical analyzes were not possible once they would require original data from other studies, as well as a database equivalent to running comparison methods parametric or non-parametric tests, thus we only inferred with these data a statistical tendency to the difference between all manometric parameters studied for all articles.

## Conclusion

Further comparative studies in healthy based on the London Protocol under a water perfusion high-resolution system may allow, especially in those countries where solid-state equipment is not available, reproducibility and dataset expansion to disease comparisons research. Nevertheless, the regionalization of certain manometric findings can also be verified and must be consider, similar to SS system comparisons, depending on the potential impact of different software and configurations probes in use as well as technical protocols.

Furthermore, 3D HRM can provide complementary and more accurate data for understanding the physiology and pathophysiology mechanism of anorectal disorders and contribute to tailored therapy, thus additional and broader investigation will be important to support this.

### Supplementary Information

**Supplementary Material 1.**

## Data Availability

All data are provided in the manuscript and data files, with corresponding figures and tables attached,as well as in supplementary information (supplementary figures and tables) attached.
