# Correction to: London protocol under water-perfused HRM in a healthy population, towards novel 3D manometric parameters in an evaluation of anorectal functional disorders

**DOI:** 10.1186/s12876-024-03327-3

**Published:** 2024-07-27

**Authors:** Alexandre Anefalos, Carlos Augusto Real Martinez, Claudio Saddy Rodrigues Coy

**Affiliations:** Department of Surgery, FCM, State University of Campinas-UNICAMP, Campinas, SP Brazil

**Correction: BMC Gastroenterol 24, 127 (2024).**

10.1186/s12876-024-03207-w

Following publication of the original article it was reported that there were errors in Tables 2 and 6 and Supplementary Table 3.

Regarding Table 2 and Supplementary Table 3, for the variable long squeeze PV (3/3) 10^4^mmHg².cm of female one value was mistakenly included in the original in the scale of 10^3^ instead of 10^4^, which misled the analysis of the maximum value and the mean, although the median and interquartile range were not affected. The original standard deviation reported was 18.5, while the correct value is 5.6. Additionally, the mean value, originally reported as 11.8, is 8.3.

The corrected Table 2 and Supplementary Table 3 are given in this Correction with the corrected values highlighted in italics in Table 2.

**Table 2:** Squeeze manometric parameters comparing 25 healthy (female x male)

Squeeze manometric parameters	Female (25) Mean (SD) Med (IQR)		Male (25)		*p*
			Mean (SD)	Med (IQR)	
London Protocol parameters					
**Maximum incremental pressure squeeze (mmHg);** short squeeze	108.7 (42.7)	102.5 [83.6;126.2]	143 (50.5)	146.3 [114.0;168.8]	**< 0.05**
**Complementary parameters**					
**Mean pressure (mmHg);** short squeeze	117.0 (40.6)	107.8 [99.6;138.0]	155.1 (39.4)	157.2 [131.6;177.6]	**< 0.01**
**Maximum absolute squeeze pressure (mmHg);** short squeeze	169.9 (44.0)	168.5 [142.8;183.5]	205.7 (45.2)	211.6 [180.0;239.6]	**< 0.01**
**Fatigue rate (mmHg);** long squeeze	-74.9 (46.4)	-69.8 [-91.8; -47.5]	-60.5 (77.5)	-71.3 [-95.4; -13.8]	0.64
**Fatigue rate index (min);** long squeeze	1.6 (2.2)	0.8 [0.6;1.5]	2.0 (4.2)	1.0 [0.7;1.5]	0.42
**Capacity to sustain (%);** long squeeze	71.60 (14.8)	73.4 [61.0;80.0]	79.80 (22.1)	74.2 [66.9;93.6]	0.24
**3D parameters**					
**Short squeeze PV (10** ^4^ **mmHg².cm)**	21.0 (13.1)	18.8 [13.5;23.9]	36.2 (16.4)	30.9 [26.0;49.9]	**< 0.01**
**Highest-pressure asymmetry (%); short squeeze**	15.8 (5.6)	16.0 [10.6;18.1]	14.1 (4.9)	13.5 [10.4;17.2]	0.27
**Lowest pressure asymmetry (%); short squeeze**	18.8 (9.2)	17.2 [11.4;23.3]	12.9 (4.5)	12.6 [9.7;15.1]	**< 0.05**
**Long squeeze PV (1/3) 10** ^4^ **mmHg².cm**	11.8 (7.9)	9.9 [7.7;13.7]	19.0 (10.5)	15.4 [12.3;24.8]	**< 0.01**
**Long squeeze PV (2/3) 10** ^4^ **mmHg².cm**	9.9 (7.2)	7.7 [4.4;13.1]	16.4 (10.5)	12.2 [9.1;24.7]]	**< 0.01**
**Long squeeze PV (3/3) 10** ^4^ **mmHg².cm**	*8.3 (5.6)*	7.2 [4.0;11.3]	15.1 (10.7)	11.0 [9.1;20.5]	**< 0.05**

*SD* Standard deviation, *Med* Median, IQR Interquartile range, *PV* Pressure-volume

Bold values indicate statistically significant (*p* < 0.05)

In the fourth column of Table 6 the name ‘Viebig’ was incorrectly given as ‘Vierbig’.

The original article has been corrected.

### Electronic supplementary material

Below is the link to the electronic supplementary material.

Supplementary Material 1

