# Supplementary material for: London Protocol under water-perfused HRM in a healthy population, towards novel 3D manometric parameters in an evaluation of anorectal functional disorders
Source: BMC Gastroenterol. 2024 Apr 4;24:127. doi: 10.1186/s12876-024-03207-w (PMC10996243; doi:10.1186/s12876-024-03207-w)
Supplement: Supplementary file 1 — Supplementary Material 1. [file 12876_2024_3207_MOESM1_ESM.pdf]

London Protocol under water-perfused HRM in a healthy population, towards novel 3D manometric parameters in an evaluation of anorectal functional disorders.

Supplementary Materials

a. Supplementary Figures (SF)

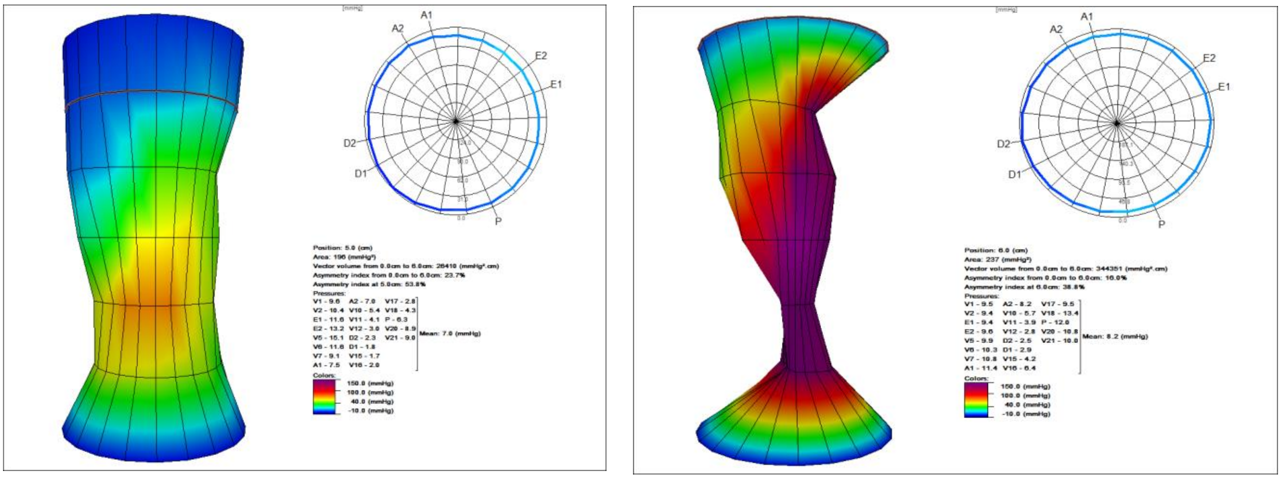

(a) **(b)**

SF 1. Example of resting PV (a) and short squeeze PV (b)

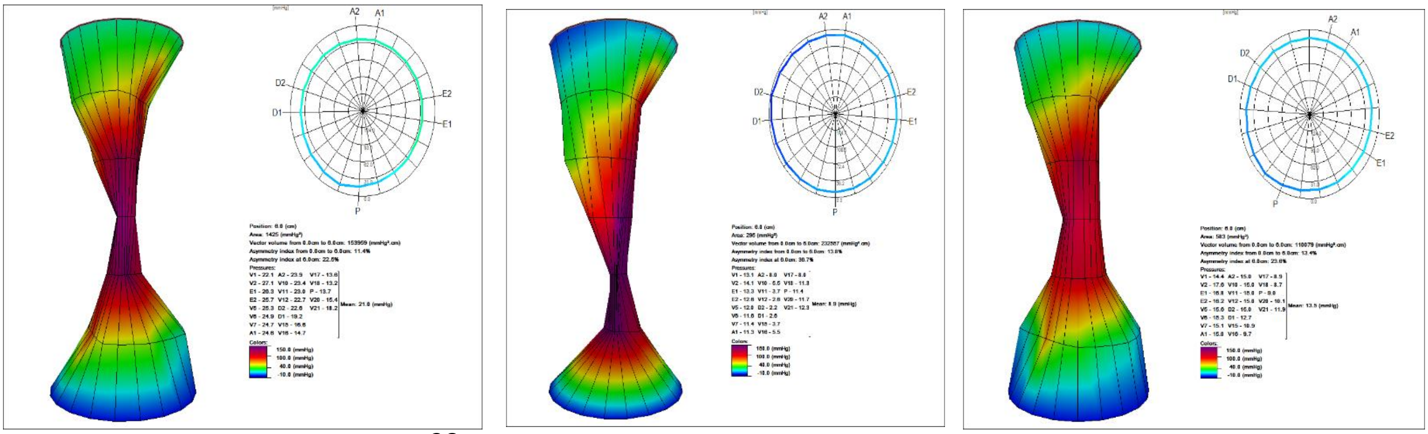

(a) **(b)** **(c)**

SF 2 Example of long squeeze PV: 1/3 (a), 2/3 (b) and 3/3 (c)

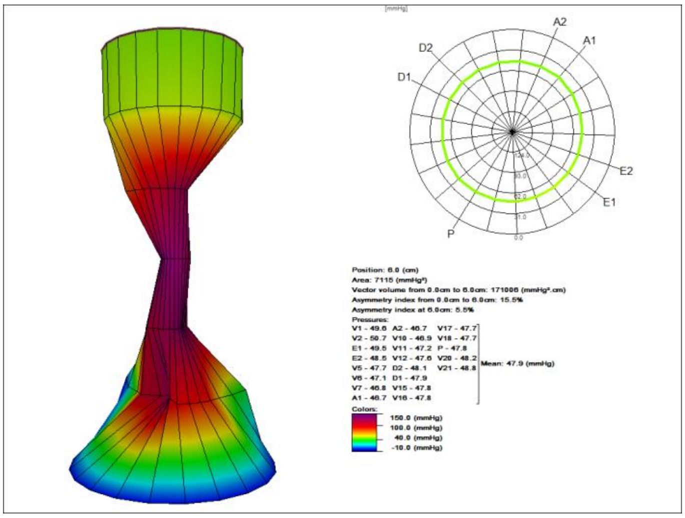

SF 3 Example of anal canal PV in cough

b. Supplementary Tables (ST)

Table ST1 Resting manometric parameters in 25 healthy females

Resting manometric parameters (females)	Mean (SD)	Med (IQR)	Min; Max	95% CI	5th; 95th
		**London Protocol**			
**Mean maximum pressure (mmHg)**	61.1 (16.3)	59.9[51.9;74.0]	[30.5; 95.7]	[54.63;67.62]	[31.90; 77.90]
		**Complementary parameters**			
**Mean pressure (mmHg)**	38.4 (9.6)	38.9[30.7;46.0]	[22.3;59.1]	[34.55;42.20]	[23.06;51.04]
**Functional anal canal length (cm)**	3.5 (1.0)	3.6[2.7;4.4]	[1.6;5.2]	[3.10;3.92]	[1.78;4.90]
		**3D parameters**			
**Resting PV (10^4mmHg^2.cm)**	2.4 (1.5)	2.0[1.1;3.3]	[0.6;6.1]	[1.81;2.99]	[0.69;4.38]
**Highest pressure asymmetry (%)**	26.3(9.8)	23.9[19.3;29.9]	[11.2;51.7]	[22.40;30.20]	[16.44;44.58]
**Lowest pressure asymmetry (%)**	27.1(13.0)	25.4[19.4;32.4]	[6.6;56.7]	[21.87;32.24]	[9.80;51.30]

SD (standard deviation); Med (median); IQR (interquartile range); PV (pressure-volume); CI (confidence interval)

Table ST2 Resting manometric parameters in 25 healthy males

Resting manometric parameters (males)	Mean (SD)	Med (IQR)	Min; Max	95% CI	5th; 95th
		**London Protocol**			
**Mean maximum pressure (mmHg)**	62.8 (14.7)	60.9[50.7;75.3]	[39.3; 87.2]	[48.1;77.5]	[43.0; 86.44]
		**Complementary parameters**			
**Mean pressure (mmHg)**	42.1 (9.2)	40.0[35.4;45.1]	[26.9;61.6]	[38.46;45.81]	[30.10;60.24]
**Functional anal canal length (cm)**	3.9 (0.8)	3.9[3.3;4.6]	[1.9;5.3]	[3.57;4.24]	[2.82;4.98]
		**3D parameters**			
**Resting PV ($10^4 mmHg^2.cm$)**	2.9 (1.6)	2.6[1.7;3.6]	[0.6;6.5]	[2.27;3.58]	[0.97;6.09]
**Highest pressure asymmetry (%)**	27.3(9.6)	26.8[21.1;32.5]	[8.4;46.4]	[23.48;31.09]	[14.88;43.18]
**Lowest pressure asymmetry (%)**	23.2(10.0)	22.6[17.5;28.2]	[5.9;50.8]	[19.24;27.20]	[10.14;40.76]

SD (standard deviation); Med (median); IQR (interquartile range); PV (pressure-volume); CI (confidence interval)

Table ST3 Squeeze manometric parameters in 25 healthy females

Squeeze manometric parameters (females)	Mean (SD)	Med (IQR)	Min; Max	95% CI	5th; 95th
		**London Protocol**			
**Maximum incremental pressure squeeze (mmHg); short squeeze**	108.7 (42.7)	102.5 [83.6;126.2]	[11.6; 208.6]	[91.73; 125.75]	[64.46; 178.80]
		**Complementary parameters**			
**Mean pressure (mmHg); short squeeze**	117.0 (40.6)	107.8 [99.6;138.0]	[40.8; 204.7]	[100.78; 133.14]	[63.02; 201.26]
**Maximum absolute squeeze pressure (mmHg); short squeeze**	169.9 (44.0)	168.5 [142.8;183.5]	[71.1; 263.8]	[152.31; 187.42]	[114.90; 246.64]
**Fatigue rate (mmHg); long squeeze**	-74.9 (46.4)	-69.8 [-91.8; -47.5]	[-201.10; -1.4]	[-93.37; - 56,40]	[-155.78; -10.22]
**Fatigue rate index (min); long squeeze**	1.6 (2.2)	0.8 [0.6;1.5]	[0.3; 10.5]	[0.70; 2.48]	[0.40; 5.66]
**Capacity to sustain (%); long squeeze**	71.60 (14.8)	73.4 [61.0;80.0]	[47.1; 98.6]	[65.70; 77.53]	[49.14; 95.80]
		**3D parameters**			
**Short squeeze PV (10^4mmHg^2.cm)**	21.0 (13.1)	18.8 [13.5;23.9]	[2.0; 56.4]	[15.80; 26.28]	[6.23; 48.61]
**Highest-pressure asymmetry (%); short squeeze**	15.8(5.6)	16.0 [10.6;18.1]	[6.2; 27.8]	[13.51; 18.01]	[8.60; 26.16]
**Lowest pressure asymmetry (%); short squeeze**	18.8(9.2)	17.2 [11.4;23.3]	[7.3; 42.9]	[15.10; 22.41]	[7.98; 33.22]
**Long squeeze PV (1/3) 10^4mmHg^2.cm**	11.8 (7.9)	9.9 [7.7;13.7]	[1.0; 33.7]	[8.65; 14.97]	[3.17; 27.92]
**Long squeeze (2/3) 10^4mmHg^2.cm**	9.9 (7.2)	7.7 [4.4;13.1]	[0.7; 30.8]	[6.97; 12.73]	[2.58; 22.73]
**Long squeeze PV (3/3) 10^4mmHg^2.cm**	8.3 (5.6)	7.2 [4.0;11.3]	[0.9; 22.4]	[6.02; 10.62]	[1.83; 18.85]

SD (standard deviation); Med (median); IQR (interquartile range); PV (pressure-volume); CI (confidence interval)

Table ST4 Squeeze manometric parameters in 25 healthy males

Squeeze manometric parameters (females)	Mean (SD)	Med (IQR)	Min; Max	95% CI	5th; 95th
		**London Protocol**			
**Maximum incremental pressure squeeze (mmHg); short squeeze**	143 (50.5)	146.3 [114.0;168.8]	[41.9; 262.0]	[122.84; 163.09]	[64.72; 216.18]
		**Complementary parameters**			
**Mean pressure (mmHg); short squeeze**	155.1 (39.4)	157.2 [131.6;177.6]	[87.3; 256.9]	[139.40; 170.84]	[95.28; 201.82]
**Maximum absolute squeeze pressure (mmHg); short squeeze**	205.7 (45.2)	211.6 [180.0;239.6]	[123.4; 301.3]	[187.72; 223.74]	[128.86; 269.30]
**Fatigue rate (mmHg); long squeeze**	-60.5 (77.5)	-71.3 [-95.4; -13.8]	[-267.9; 132.8]	[-91.41; -29.59]	[-137.88; 83.82]
**Fatigue rate index (min); long squeeze**	2.0 (4.2)	1.0 [0.7;1.5]	[0.4; 20.5]	[0.35; 3.68]	[0.41; 2.87]
**Capacity to sustain (%); long squeeze**	79.80 (22.1)	74.2 [66.9;93.6]	[38.4; 141.3]	[70.97; 88.58]	[51.78; 120.46]
		**3D parameters**			
**Short squeeze PV (10^4mmHg^2.cm)**	36.2 (16.4)	30.9 [26.0;49.9]	[9.5; 66.6]	[29.68; 42.74]	[13.54; 63.72]
**Highest-pressure asymmetry (%); short squeeze**	14.1(4.9)	13.5 [10.4;17.2]	[6.6; 25.4]	[12.13; 16.06]	[7.72; 22.24]
**Lowest pressure asymmetry (%); short squeeze**	12.9(4.5)	12.6 [9.7;15.1]	[5.4; 24.5]	[11.15; 14.71]	[6.56; 21.06]
**Long squeeze PV (1/3) 10^4mmHg^2.cm**	19.0 (10.5)	15.4 [12.3;24.8]	[6.0; 43.6]	[14.85; 23.21]	[6.40; 37.66]
**Long squeeze PV (2/3) 10^4mmHg^2.cm**	16.4 (10.5)	12.2 [9.1;24.7]]	[4.8; 44.0]	[12.22; 20.61]	[5.62; 36.69]
**Long squeeze PV (3/3) 10^4mmHg^2.cm**	15.1 (10.7)	11.0 [9.1;20.5]	[4.3; 43.9]	[10.85; 19.36]	[5.13; 38.63]

SD (standard deviation); Med (median); IQR (interquartile range); PV (pressure-volume); CI (confidence interval)

Table ST5 Cough manometric parameters in 25 healthy females

Cough manometric parameters (females)	Mean (SD)	Med (IQR)	Min; Max	95% CI	5th; 95th
**London Protocol**					
**Maximum pressure anal canal (mmHg)**	132 (31.6)	134.6 [127.6;155.0]	[62.3; 182.6	[120.60; 145.83]	[79.42; 173.82]
**Maximum pressure rectum (mmHg)**	65.5 (23.8)	65.0 [47.0;79.3]	[24.1; 117.1]	[56.06; 75.02]	[34.74; 109.42]
**Complementary parameters**					
**Anorectal gradient pressure (mmHg)**	67.7 (22.1)	62.6 [48.6;83.9]	[31.4; 103.3]	[58.84; 76.46]	[37.56; 100.22]
**3D parameters**					
**Anal canal PV in cough (10^{4}mmHg^2.cm)**	11.0 (4.8)	10.6 [8.3;14.0]	[3.6; 21.0]	[9.08; 12.94]	[3.77; 18.65]
**Highest pressure asymmetry (%); anal canal**	14.4(5.9)	13.9 [10.0;16.9]	[5.1; 26.7]	[12.00; 16.71]	[6.58; 26.0]
**Lowest pressure asymmetry (%); anal canal**	14.7(4.9)	14.9 [12.6;18.1]	[6.3; 24.7]	[12.79; 16.66]	[6.54; 21.94]

SD (standard deviation); Med (median); IQR (interquartile range); PV (pressure-volume); CI (confidence interval)

Table ST6 Cough manometric parameters in 25 healthy males

Cough manometric parameters (males)	Mean (SD)	Med (IQR)	Min; Max	95% CI	5th; 95th
**London Protocol**					
**Maximum pressure anal canal (mmHg)**	150.7 (28.8)	150.2 [124.9;171.6]	[95.9; 199.4]	[139.20; 162.21	[106.66; 195.96]
**Maximum pressure rectum (mmHg)**	87.0 (27.3)	82.2 [72.5;95.5]	[43.9; 172.5]	[76.07; 97.85	[53.20; 132.22]
**Complementary parameters**					
**Anorectal gradient pressure (mmHg)**	63.7 (26.8)	63.1 [55.7;71.9]	[5.6;138.1]	[53.05; 74.43]	[25.94; 106.94]
**3D parameters**					
**Anal canal PV in cough (10^4mmHg^2.cm)**	15.1 (5.2)	15.3 [11.6;18.2]	[6.4;26.7]	[13.03; 17.16]	[7.41; 22.71]
**Highest pressure asymmetry (%); anal canal**	16.0(6.7)	14.9 [10.8;20.3]	[7.6;33.2]	[13.31; 18.66]	[7.80; 27.28]
**Lowest pressure asymmetry (%); anal canal**	12.5(4.0)	11.7 [8.9;15.0]	[7.0;21.2]	[10.89; 14.12]	[7.68; 19.40]

SD (standard deviation); Med (median); IQR (interquartile range); PV (pressure-volume); CI (confidence interval)

Table ST7 Push manometric parameters in 25 healthy females

Push manometric parameters (females)	Mean (SD)	Med (IQR)	Min; Max	95% CI	5th; 95th
**London Protocol**					
**Maximum pressure anal canal (mmHg)**	47.4 (19.0)	43.9 [35.7;53.9]	[14.2; 87.2]	[39.87; 54.98]	[25.88; 84.22]
**Maximum pressure rectum (mmHg)**	27.9 (20.5)	23.9 [19.0;33.2]	[6.8;112.5]	[19.77; 36.07]	[9.84; 45.66]
**Complementary parameters**					
**Rectum-anal gradient pressure (mmHg)**	-16.1 (22.5)	-17.9 [-24.2; -1.5]	[-66.4; 27.9]	[-25.03; -7.08]	[-55.72; 24.16]
**Anal canal relaxation percent (%)**	6.4 (36.0)	10.2 [-22.0;36.4]	[-79.7; 70.1]	[-7.94; -20.77]	[-50,24; 50.64]

SD (standard deviation); Med (median); IQR (interquartile range); PV (pressure-volume); CI (confidence interval)

Table ST8 Push manometric parameters in 25 healthy males

Push manometric parameters (males)	Mean (SD)	Med (IQR)	Min; Max	95% CI	5th; 95th
		**London Protocol**			
**Maximum pressure anal canal (mmHg)**	61.3 (26.5)	51.3 [46.7;71.1]	[11.3; 128.8]	[50.79; 71.90]	[31.82; 115.08]
**Maximum pressure rectum (mmHg)**	42.5 (18.5)	34.9 [31.1;54.1]	[20.0;91.5]	[35.12; 49.83]	[24.48; 72.86]
		**Complementary parameters**			
**Rectum-anal gradient pressure (mmHg)**	-22.9 (13.2)	-20.2 [-31.4; -13.9]	[-55.3; -4.1]	[-28.12; -17.62]	[-40.32; -6.20]
**Anal canal relaxation percent (%)**	-23.7 (54.1)	-5.7 [-48.8;10.2]	[-157.8; 36.7]	[-45.26; -2.15]	[-145.72; 28.74]

SD (standard deviation); Med (median); IQR (interquartile range); PV (pressure-volume); CI (confidence interval)

Table ST9 RAIR manometric parameters (25 females, 25 males and comparison)

RAIR manometric complementary parameter	Mean (SD)	Med (IQR)	Min; Max	95% CI	5th; 95th	p
**Anal canal relaxation (%)**						
**Female**	36.8 (15.8)	33.3 [25.7;44.9]	[10.90;73.0]	[30.48;43,11]	[17.92;68.50]	0.70
**Male**	38.4 (13.9)	39.4 [29.5;45.4]	[8.2;61.4]	[32.87;43,98]	[32.87;43.98]	

SD (standard deviation); Med (median); IQR (interquartile range); PV (pressure-volume); CI (confidence interval)

Table ST10 Rectal sensory thresholds parameters (25 females, 25 males and comparison)

Rectal sensory thresholds parameters	Mean (SD)	Med (IQR)	Min; Max	95% CI	5th; 95th	p
			**London Protocol**			
**First sensation volume (ml)**						
**Female**	22.9 (17.9)	16.0[10.0;32.0]	[6.0;78]	[15.76;30.08]	[8.0;50.0]	0.35
**Male**	20.70 (20.40)	12.0[6.0;22.0]	[4.0;88]	[12.60;28.84]	[5.20;50.0]	
**Desire to defaecate volume (ml)**						
**Female**	38.5 (19.70)	34.0[23.0;48.0]	[14.0;100.0]	[30.62;46.34]	[16.0;68.0]	0.77
**Male**	39.20 (24.0)	34.0 [20.0;50.0]	[12.0;100.0]	[29.60;48.71]	[12.40; 86.0]	
**Maximum tolerated volume(ml)**						
**Female**	141.4 (53.10)	132.0[106.0;164.0]	[70.0;270.0]	[120.24;162.56]	[73.0;243.80]	0.57
**Male**	133.4 (44.3)	125.0[100.0;140.0]	[84.0;240.0]	[115.75;151.05]	[86.40;226.0]	

SD (standard deviation); Med (median); IQR (interquartile range); PV (pressure-volume); CI (confidence interval)

London Protocol under water-perfused HRM in a healthy population, towards novel 3D manometric parameters in an evaluation of anorectal functional disorders.
