# Supplementary material for: Correction to: London protocol under water-perfused HRM in a healthy population, towards novel 3D manometric parameters in an evaluation of anorectal functional disorders
Source: BMC Gastroenterol. 2024 Jul 27;24:236. doi: 10.1186/s12876-024-03327-3 (PMC11282614; doi:10.1186/s12876-024-03327-3)
Supplement: Supplementary file 1 — Supplementary Material 1 [file 12876_2024_3327_MOESM1_ESM.pdf]

London Protocol under water-perfused HRM in a healthy population, towards novel 3D manometric parameters in an evaluation of anorectal functional disorders.

Table ST3 Squeeze manometric parameters in 25 healthy females

Squeeze manometric parameters (females)	Mean (SD)	Med (IQR)	Min; Max	95% CI	5th; 95th
**London Protocol**					
**Maximum incremental pressure squeeze (mmHg); short squeeze**	108.7 (42.7)	102.5 [83.6;126.2]	[11.6; 208.6]	[91.73; 125.75]	[64.46; 178.80]
**Complementary parameters**					
**Mean pressure (mmHg); short squeeze**	117.0 (40.6)	107.8 [99.6;138.0]	[40.8; 204.7]	[100.78; 133.14]	[63.02; 201.26]
**Maximum absolute squeeze pressure (mmHg); short squeeze**	169.9 (44.0)	168.5 [142.8;183.5]	[71.1; 263.8]	[152.31; 187.42]	[114.90; 246.64]
**Fatigue rate (mmHg); long squeeze**	-74.9 (46.4)	-69.8 [-91.8; -47.5]	[-201.10; -1.4]	[-93.37; - 56,40]	[-155.78; -10.22]
**Fatigue rate index (min); long squeeze**	1.6 (2.2)	0.8 [0.6;1.5]	[0.3; 10.5]	[0.70; 2.48]	[0.40; 5.66]
**Capacity to sustain (%); long squeeze**	71.60 (14.8)	73.4 [61.0;80.0]	[47.1; 98.6]	[65.70; 77.53]	[49.14; 95.80]
**3D parameters**					
**Short squeeze PV (10^4mmHg^2.cm)**	21.0 (13.1)	18.8 [13.5;23.9]	[2.0; 56.4]	[15.80; 26.28]	[6.23; 48.61]
**Highest-pressure asymmetry (%); short squeeze**	15.8(5.6)	16.0 [10.6;18.1]	[6.2; 27.8]	[13.51; 18.01]	[8.60; 26.16]
**Lowest pressure asymmetry (%); short squeeze**	18.8(9.2)	17.2 [11.4;23.3]	[7.3; 42.9]	[15.10; 22.41]	[7.98; 33.22]
**Long squeeze PV (1/3) 10^4mmHg^2.cm**	11.8 (7.9)	9.9 [7.7;13.7]	[1.0; 33.7]	[8.65; 14.97]	[3.17; 27.92]
**Long squeeze (2/3) 10^4mmHg^2.cm**	9.9 (7.2)	7.7 [4.4;13.1]	[0.7; 30.8]	[6.97; 12.73]	[2.58; 22.73]
**Long squeeze PV (3/3) 10^4mmHg^2.cm**	8.3 (5.6)	7.2 [4.0;11.3]	[0.9; 22.4]	[6.02; 10.62]	[1.83; 18.85]

SD (standard deviation); Med (median); IQR (interquartile range); PV (pressure-volume); CI (confidence interval)
